# Threatened by mining, polymetallic nodules are required to preserve abyssal epifauna

**DOI:** 10.1038/srep26808

**Published:** 2016-06-01

**Authors:** Ann Vanreusel, Ana Hilario, Pedro A. Ribeiro, Lenaick Menot, Pedro Martínez Arbizu

**Affiliations:** 1 Marine Biology, Ghent University, 9000 Ghent, Belgium; 2 Biology Department & Centre for Environmental and Marine Studies, University of Aveiro, 3810 Aveiro, Portugal; 3 MARE- Marine and Environmental Sciences Centre. Department of Oceanography and Fisheries of the University of the Azores, 9901-862 Horta, Portugal; 4 Okeanos- R&D Center, University of the Azores, 9901-862 Horta, Portugal; 5 Deep-Sea Environment Laboratory, Ifremer, Centre de Bretagne, 29280 Plouzané, France; 6 Senckenberg am Meer, Abt. DZMB, 26382 Wilhelmshaven, Germany

## Abstract

Polymetallic nodule mining at abyssal depths in the Clarion Clipperton Fracture Zone (Eastern Central Pacific) will impact one of the most remote and least known environments on Earth. Since vast areas are being targeted by concession holders for future mining, large-scale effects of these activities are expected. Hence, insight into the fauna associated with nodules is crucial to support effective environmental management. In this study video surveys were used to compare the epifauna from sites with contrasting nodule coverage in four license areas. Results showed that epifaunal densities are more than two times higher at dense nodule coverage (>25 versus ≤10 individuals per 100 m^2^), and that taxa such as alcyonacean and antipatharian corals are virtually absent from nodule-free areas. Furthermore, surveys conducted along tracks from trawling or experimental mining simulations up to 37 years old, suggest that the removal of epifauna is almost complete and that its full recovery is slow. By highlighting the importance of nodules for the epifaunal biodiversity of this abyssal area, we urge for cautious consideration of the criteria for determining future preservation zones.

Polymetallic nodules are found at the surface of soft deep-sea bottoms at abyssal depths. Large areas in different parts of the Pacific and Indian oceans are known to have high concentrations of these nodular deposits^1^,^2^. Even though very little is known on the biodiversity associated with nodules in these highly remote places^3^,^4^,^5^, vast areas are being targeted by concession holders for future mining^6^. Despite the present lack of knowledge, large-scale harmful effects of these activities are expected^7^. Since the International Seabed Authority (ISA) aims to develop a regulatory framework for mineral exploitation in the area beyond national jurisdiction in the near future^8^,^9^, an improved knowledge of the fauna associated with nodules is crucial for establishing mining regulations and procedures^6^,^7^,^8^.

Rich in copper, cobalt, nickel and manganese, polymetallic nodules have been receiving varying levels of attention from governments and industry, depending on the prevailing socio-economic and political settings^8^. The recognition of the potentially high economic value of mineral deposits in areas beyond national jurisdiction (the Area) led in 1982, under the UN convention on the Law of the Sea (UNCLOS), to the implementation of ISA, an organization that is responsible for the management of these resources, as well as the conservation and protection of the marine environment and its flora and fauna from mining activities (Article 145 UNCLOS)^8^. Since then, and with increasing frequency in the past few years, different organizations have applied for mining licenses in the Clarion Clipperton Fracture Zone (CCZ), the main area of worldwide interest in terms of minable minerals^8^. Contractors are required to survey the biota of their license area and to evaluate the possible impact of their planned mining activities on the environment. Since the first of these exploration contracts expires in 2016, a regulatory framework for mineral exploitation in the Area needs to be implemented urgently^9^,^10^. In this context the international deep-sea research community has recognized the need for large scale investigations^8^,^11^,^12^, complementary to the exploration surveys produced by the various contractors, which are spatially limited to their license areas. Considering the basin-wide impact that multiple mining operations will exert, understanding the large-scale distribution of species and communities is of paramount importance for their effective preservation.

Four license areas (Fig. 1), covering approximately 1300 km in an east-west direction along the eastern CCZ, plus one of the nine Areas of Particular Environmental Interest (APEI no. #3)^6^,^8^,^9^ targeted by ISA for preservation, were visited during the EcoResponse cruise (March–April 2015) on board the RV Sonne. Standardized video transects, 17 in total, were performed one meter above the seafloor using the Remote Operated Vehicle (ROV) Kiel 6000 to identify composition and densities of both sessile and mobile megabenthic epifauna (excluding fish, crustaceans and large protozoans) in areas with dense nodule concentrations (>15% cover) and areas with very few or no obvious surface nodules (<1%) (Fig. 2).

Epifaunal composition and densities were also quantified for two areas, where experimental mining simulations were performed more than two decades ago (20 and 37 years) (Fig. 2)^13^,^14^, as well as in two areas with recent trawl tracks (eight months and three years old). Comparison of the epifaunal assemblages identified from these transects enabled us to identify the importance of nodules for local biodiversity, validating the impact of nodule removal, estimating the recovery at decadal time-scales and gathering preliminary data on one of the APEIs for which virtually nothing is known^8^.

## Results and Discussion

Sessile metazoan epifauna on nodule transects comprised 8 major groups, representing Hydrozoa, Anthozoa (Actiniaria, Alcyonacea, Antipatharia, Corallimorpharia, Ceriantharia), Echinodermata (Crinoidea) and Porifera (Hexactinellida). Species-level identification of deep-sea taxa from video is notably difficult. Although dozens of morphotypes were identified in the surveys, we kept epifaunal identifications to higher taxonomic ranks (Class or Order). Morphological and molecular analyses are currently being conducted on voucher specimens collected during the ROV dives to validate video observations as well as to determine true species diversity and population connectivity.

Overall densities of sessile taxa in nodule areas varied between 14 and 30 individuals per 100 m^2^ (Fig. 3). In nodule areas, Anthozoans, mainly Alcyonacea and Actiniaria, were the most abundant sessile group with over 63% and up to 91% of the observations in each transect, followed by sponges (Porifera) representing 6% to 36% of the sessile fauna. Densities were lower in areas with no or only rare surface nodules, never exceeding eight individuals per 100 m^2^ (Fig. 3). Most of the recorded higher taxa were represented in both nodule-rich and nodule-free areas. Alcyonacea, Antipatharia, Actiniaria and Porifera were represented by several morphotypes, many of which were growing on nodules (Fig. 4).

Mobile epifauna was represented in both nodule-rich and nodule-free areas by echinoderms belonging to four groups: Holothuroidea, Ophiuroidea, Echinoidea and Asteroidea. Densities varied between 4–15 individuals per 100 m^2^ among nodule-rich sites, largely due to the contribution of ophiuroids. Densities of the mobile epifauna were more than two times lower (1–3 ind./100 m^2^) on nodule-free sites compared to nodule covered transects performed in the same geographical area, with a particularly large decrease (>50%) in ophiuroids and echinoids.

Video transects along recent tracks (eight months old from a dredge in a nodule site in the GSR license area and approximately three years old from an epibenthic sledge track in a nodule-free IFREMER area) revealed an almost complete depletion of sessile epifauna, with only a few actiniarians (0.2 ind./100 m^2^) inhabiting the sideward displaced nodules and sediment. Echinoids were the most successful colonizers of the three-year old track showing densities nine times higher (1.4 ind./100 m^2^) than on the surrounding undisturbed, nodule-free seabed (0.15 ind./100 m^2^). However, on the eight month old track of the GSR area mobile fauna (5 ind./100 m^2^) decreased by 50% or more compared to the nearby undisturbed seabed containing nodules (12–15 ind./100 m^2^).

Epifaunal communities along the two historical experimental mining tracks were also severely impoverished, in comparison to surrounding reference sites, indicating poor recolonization rates even decades after the disturbance. Sessile epifauna on the 20-year-old track located in an nodule-free site of the IOM license area^13^ was limited to very few sponges (0.4 ind./100 m^2^), whereas on the reference nodule-free transect actiniarians, alcyonaceans, antipatharians and ceriantharians were also found (overall density 6.8 ind./100 m^2^). Composition of mobile epifauna inside and outside the track was similar, but density was 50% lower inside the track (respectively 1.5 and 3 ind./100 m^2^). The 37-year-old track on a nodule-rich section of the IFREMER license area^14^ contrasted even more with its corresponding reference site. Sessile epifauna dropped from 24.1 outside to just 3.6 individuals per 100 m^2^ inside the track, and consisted mainly of actiniarians. Mobile epifauna inside the track was 70% less abundant (1 ind./100 m^2^) than on the neighboring undisturbed seabed (4 ind./100 m^2^) and was only represented by echinoids.

**Figure 1:**
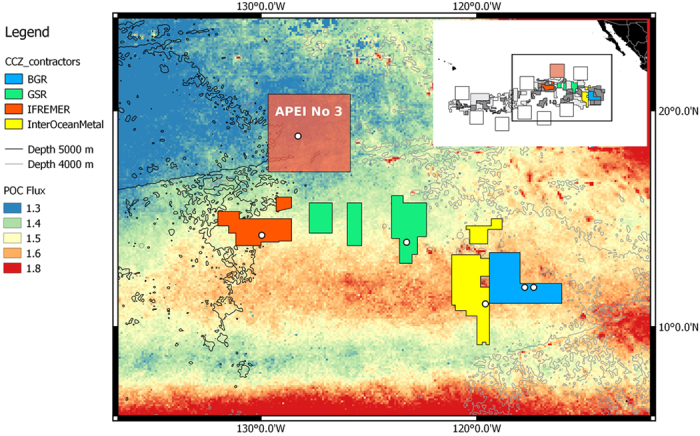
Map of the CCZ highlighting four license areas and APEI no. 3, surveyed during the Ecoresponse cruise. White circles indicate the video transect sites. Background colors represent the upper limit in POC flux (mg C_org_ m^−2^ d^−1^)^16^. The inset map shows the location of the surveyed areas (in color) relative to all other reserved areas for mining (in gray) and APEIs (open squares) in the CCZ. Map was generated using the software Quantum GIS 2.8 (www.qgis.org) Copyright QGIS Development Team. The base maps used are: Shapefile of the contractor areas and APEIs kindly provided by ISA, Kingston, Jamaica.

**Figure 3:**
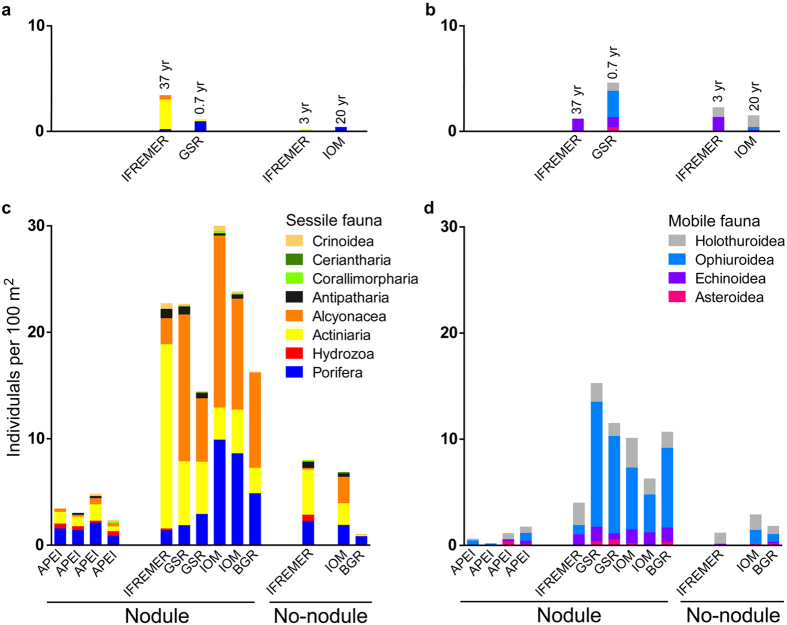
Densities (ind./100 m^2^) of sessile (**a,c**) and mobile (**b,d**) epifauna for separate ROV transects from areas rich in nodules, and nodule-free areas. (**a,b)** Densities from ROV transects experimentally disturbed areas of various age; (**c,d)** Densities from undisturbed areas.

**Figure 4:**
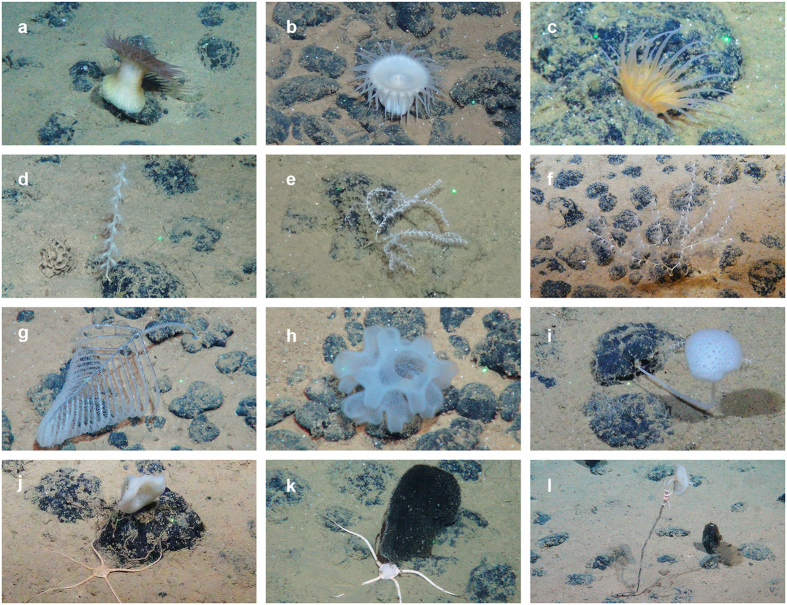
Examples of sessile epifauna associated with nodules. (**a–c)** actiniarians; (**d–f)** alcyonacean corals; (**g)** antipatharian coral; (**h–l)** hexactinellid sponges. Copyright: ROV Kiel 6000 Team/ GEOMAR Kiel.

In comparison to nodule fields in the central CCZ, all four video transects performed in the area surveyed at the center of APEI no #3, which is also nodule-rich, showed low densities of both sessile (2–5 ind./100 m^2^) and mobile fauna (<3 ind./100 m^2^). This area is situated below the most oligotrophic surface waters of this oceanic region (Fig. 1), at the northern edge of the Northern equatorial surface current^15^, which may explain the low numbers^4^ compared to the more southern areas in the CCZ, where spring blooms are more prominent and higher POC fluxes are expected^16^ especially in the eastern part of the surveyed area.

Polymetallic nodule mining will impose profound effects on abyssal ecosystems due to the disturbance of top sediment layers, together with the resuspension and subsequent deposition of sediments^17^,^18^. Particularly drastic and possibly permanent damage, however, will be caused by the removal of a hard substrate which, according to the data presented here, will definitely lead to significant biodiversity loss, some of which may never recover considering that nodules only grow a few mm per million years^1^, and that some taxa such as alcyonacean and antipatharian corals in this area occur exclusively on hard surfaces. Although our study sites in the CCZ are influenced by different environmental settings (e.g. depth, productivity^15^,^16^,^19^), we present consistent results of lower epifauna densities in areas with less nodules compared to nodule rich areas, and slow recovery of impacted sites.

Furthermore, it is conceivable that factors promoting the presence of high densities of economically important nodules, such as low sedimentation rates and moderate bottom currents at slightly elevated parts of the abyssal seafloor^20^, also incidentally help to sustain local biodiversity of mainly suspension feeding epifauna^4^,^19^, as compared to depressions where sedimentation rates are higher and bottom currents weaker.

When considering the spatial and temporal scales of nodule mining impact on epifauna, there is a clear need for in-depth environmental management systems and mitigation strategies. Though desirable, restoration in the deep sea could be several times more costly than in shallow-water marine systems^21^ and more research is necessary to develop strategies and ascertain the environmental and social benefits of restoration efforts for the nodule fauna. Meanwhile, environmental management of nodule mining is focusing on spatial management. In addition to the delineation of nine peripheral APEIs, the ISA has instigated the contractors’ obligation to identify preservation reference zones (PRZ) in their license area. However, no criteria for these PRZs have been identified so far. Therefore, economical rather than environmental criteria may encourage contractors to allocate PRZs in economically unimportant sites of their respective areas with low nodule densities. This study provides evidence that high densities of surface nodules in the PRZs is an ultimate requirement for the preservation of abyssal biodiversity within the CCZ. Furthermore, in the worst case scenario, where the epifauna of the preservation areas is also impacted by dispersing sediment plumes from intensive mining activities all around the area^17^,^18^, either resulting in covering the fauna or clogging their feeding apparatus, the presence of nodules may still enable the recovery of the local fauna in the long term. Recent papers have advocated the importance of creating large APEIs in the periphery of the reserved area for mining^6^,^9^, however, our results also point towards the need to establish PRZs within contractor areas. Although the APEIs are crucial for the preservation of the regional biodiversity in the context of a regional management plan for the CCZ^6^,^8^,^9^, they cannot replace PRZs as they lie outside the central CCZ and were not designed to facilitate recolonization of impacted sites. It is obvious that further research is required in each of the APEIs to understand how representative they are of, and connected with, the central CCZ abyssal ecosystems^6^,^8^. Our results further suggest that the removal of nodules may have a lasting impact on the epibenthic biodiversity in the contractor areas, as hard substrate will need millions of years to restore. Even within the small scale impact of trawl tracks studied here, epifauna did not show significant recovery after decades, indicating that recovery in the deep sea is a very slow process in general. Finally, investigating the present biodiversity and understanding the reproductive biology and distribution of the most prominent taxa in the whole region is an ultimate condition for a sound environmental management plan^22^,^23^. Thanks to the habitat heterogeneity they generate, polymetallic nodules sustain some of the most diverse benthic communities on the abyssal plain^24^. Nodule mining on the CCZ will have winners and losers, and hard substrate epifaunal communities will definitely be among the losers. As a regulatory framework for the exploitation of mineral resources is being developed that should further defined the PRZs, we urge the ISA to carefully take into consideration basic environmental and ecological requirements for marine spatial planning.

## Materials and Methods

A total of 17 video transects using the ROV Kiel 6000 on board the RV Sonne were performed in four concession areas in the Eastern CCZ (11°–16°N; 117°–130°W): BGR (2 transects), IOM (4 transects), GSR (3 transects) and IFREMER (4 transects), and in one of the Northern Areas of Particular Environmental Interest (APEI no #3; 4 transects). Transects covered areas with different nodule densities and areas where nodules had been experimentally removed. The ROV was flown at a speed of approximately 0.2 m s^−1^ with the high-definition color video camera (Kongsberg OE14-500; resolution: 2 mega pixels) approximately 1 m above the seafloor.

The camera was positioned at the most minimum angle possible without viewing the ROV frame; in the case of transects in areas where nodules have been removed, the field of view was set to include the whole width of the disturbed area. Transect width was calculated using two laser pointers on the seabed 6.5 cm apart from each other. The optical resolution of the cameras enabled the reliable identification of all organisms larger than 3 cm. On both transects in the BGR the camera field of view was set at a wider angle than all other transects in nodule or non-nodule areas and, therefore, the number of smaller organisms (<5 cm) may have been underestimated. All transects were annotated in real time by the same observers using the software Ocean Floor Observation Protocol (OFOP, http://ofop.texel.com). All megabenthic epifauna (excluding fish and crustaceans) were counted and identified to the higher taxonomic level; colonial organisms were counted as single individuals. During sampling operations with the ROV in the study areas, we observed positive attraction of fish and swimming decapod crustaceans to the ROV; therefore we decided to exclude them from the quantitative analysis of the video transects. Other authors have also questioned the validity of video transects for deep-sea demersal fish^25^.

As transects length was not uniform (varying from 200 to 800 m) the density of each taxonomic group was standardized by area (individuals per 100 m^2^). For this reason we did not compare measures for taxa richness between transects, but only composition at higher taxonomic level and densities. Mean nodule coverage for each transect was estimated from frame-grabs extracted every 20 m with the software OFOP. The image analyses software ImageJ (http://imagej.nih.gov) was used to quantify nodule densities in each frame-grab. The video transects from nodule-rich and nodule-free areas were performed independently in different areas across the CCZ. Logistic constraints (working with limited ship time in a remote abyssal area covering a wide geographical range) did not allow for a fully balanced and properly replicated sampling design to test the influence of nodule coverage and physical disturbances. Data analysis therefore only relied on descriptive statistics and no inferential test was carried out. Effect sizes were calculated as Cohen’s d index^26^ for comparisons of densities from the nodule-rich and nodule-free areas. Only large effect sizes (d > 0.8) were considered and discussed as it was the case for total densities of mobile and sessile fauna and densities of all taxa separate, except Hydrozoa, Ceriantharia, Coralimorpharia, Antipatharia, and Asteroidea. These taxa were all present at low densities in both nodule-rich and nodule-free areas which explains the smaller effect sizes. Transect data is available in Supplementary Table S1.

## Additional Information

**How to cite this article**: Vanreusel, A. *et al.* Threatened by mining, polymetallic nodules are required to preserve abyssal epifauna. *Sci. Rep.* **6**, 26808; doi: 10.1038/srep26808 (2016).

## Supplementary Material

Supplementary Information

## Figures and Tables

**Figure 2:**
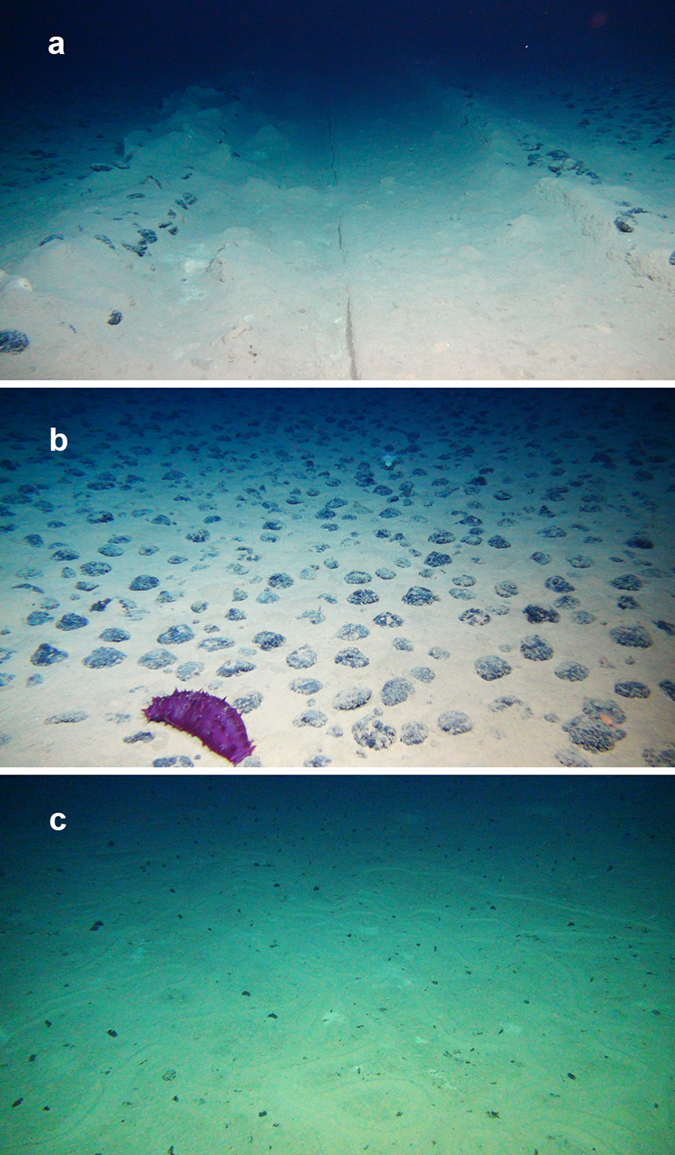
Examples of seafloor morphology: **(a)** 37-year old OMCO track (IFREMER license area); (**b)** Nodule landscape (IFREMER license area); (**c)** Nodule-free landscape (IOM area). Copyright: ROV Kiel 6000 Team/GEOMAR Kiel.
