# Supplementary material for: Threatened by mining, polymetallic nodules are required to preserve abyssal epifauna
Source: Sci Rep. 2016 Jun 1;6:26808. doi: 10.1038/srep26808 (PMC4887785; doi:10.1038/srep26808)
Supplement: Supplementary Information [file srep26808-s1.doc]

**Threatened by mining, polymetallic nodules are required to preserve abyssal epifauna**

**Ann Vanreusel^1*^**^+^**, Ana Hilario^2^**^+^**, Pedro A. Ribeiro^3,4^**^+^**, Lenaick Menot^5^**^+^ **and Pedro Martínez Arbizu^6^**^+^

^1^Marine Biology, Ghent University, 9000 Belgium.

^2^Biology Department & Centre for Environmental and Marine Studies, University of Aveiro, 3810 Aveiro, Portugal.

^3^MARE – Marine and Environmental Sciences Centre, Universidade dos Açores, Departamento de Oceanografia e Pescas, 9901-862 Horta, Açores, Portugal.

^4^IMAR- Center of the University of the Azores, Departamento de Oceanografia e Pescas, 9901-862 Horta, Portugal.

^5^Deep-Sea Environment Laboratory, Ifremer, Centre de Bretagne, 29280 Plouzané, France.

^6^Senckenberg am Meer, Abt. DZMB, 26382 Wilhelmshaven, Germany.

*ann.vanreusel@ugent.be

^+^These authors contributed equally

Supplementary Materials

Table S1-1. Transect data. The nodule coverage is presented in percentage. The age of the experimental mining simulations and trawl tracks is indicated between brackets for the transects marked with *.

| **Station** | **License area** | **Length (m)** | **Image width (m)** | **Nodule coverage % (mean ± sd)** |
| --- | --- | --- | --- | --- |
| S0239_041 | BGR | 320 | 4 | 15.0 ± 2.6 |
| S0239_064 | BGR | 870 | 4 | 0.3 ± 0.1 |
| S0239_082 | IOM | 320 | 2 | 41.3 ± 5.3 |
| S0239_082 | IOM | 410 | 2 | 44.3 ± 3.6 |
| S0239_101 | IOM | 380 | 2 | 0.5 ± 0.2 |
| S0239_101 * | IOM | 250 | 2.9 | 0 (20 yr) |
| S0239_131 | GSR | 420 | 2 | 24.4 ± 2.7 |
| S0239_141 | GSR | 450 | 2 | 21.9 ± 5.6 |
| S0239_141 * | GSR | 200 | 2.6 | 0 (0.7 yr) |
| S0239_157 | IFREMER | 400 | 2 | 29.5 ± 2.9 |
| S0239_157 * | IFREMER | 200 | 2.5 | 0 (37 yr) |
| S0239_161 | IFREMER | 450 | 2 | 0.6 ± 0.2 |
| S0239_161 * | IFREMER | 200 | 2.2 | 0 (3 yr) |
| S0239_189 | APEI | 400 | 2 | 41.6 ± 3.4 |
| S0239_189 | APEI | 350 | 2 | 43.5 ± 5.2 |
| S0239_200 | APEI | 400 | 2 | 85.4 ± 5.0 |
| S0239_200 | APEI | 400 | 2 | 92.1 1.5 |
